# Adaptive Optics (rtx1) High-Resolution Imaging of Photoreceptors and Retinal Arteries in Patients with Diabetic Retinopathy

**DOI:** 10.1155/2019/9548324

**Published:** 2019-03-17

**Authors:** Anna Zaleska-Żmijewska, Zbigniew M. Wawrzyniak, Anna Dąbrowska, Jacek P. Szaflik

**Affiliations:** ^1^Medical University of Warsaw, Department of Ophthalmology, Warsaw, Poland; ^2^SPKSO Ophthalmic Teaching Hospital, Warsaw, Poland; ^3^Warsaw University of Technology, Faculty of Electronics and Information Technology, Warsaw, Poland

## Abstract

**Background:**

Diabetic retinopathy (DR) is the leading cause of impaired vision in patients with diabetes mellitus. An adaptive optics retinal camera (rtx1™; Imagine Eyes, France) was used to capture images of cones and retinal arteries from patients with DR.

**Objective:**

Cone parameters (density, interphotoreceptor distance, and regularity) and retinal artery parameters (wall thickness, lumen diameter, WLR, and WCSA) were analyzed in 36 patients with nonproliferative DR (NPDR; 22 with mild NPDR and 14 with moderate NPDR) and in 20 healthy volunteers (the control group).

**Results:**

Cone density at 2° eccentricities was significantly lower in the DR compared to the control group (19822 ± 4342 cells/mm^2^ vs. 24722 ± 3507 cells/mm^2^, respectively). Cone density and regularity decreased with increasing severity of DR. The artery walls were significantly thicker in the DR group. The WLR and WCSA differed significantly between the DR and the control groups (WLR 0.339 ± 0.06 vs. 0.254 ± 0.04; WCSA 5567 ± 1140 vs. 4178 ± 944, respectively).

**Conclusions:**

Decreased cone regularity and density are seen in patients with mild and moderate NPDR. Abnormalities of retinal arterioles show signs of arteriolar dysfunction in DR. Retinal image analysis with the rtx1 offers a novel noninvasive measurement of early changes in the neural cells and retina vasculature in diabetic eyes.

## 1. Introduction

Diabetes is a chronic disease that causes complications in different organs, including the eye. Diabetic retinopathy (DR) is the leading cause of impaired vision worldwide [1]. It is estimated that the diabetes epidemic will result in a growing number of diabetic patients worldwide, increasing from 366 million in 2011 to 552 million in 2030 [2]. The prevalence of DR is therefore expected to be a serious problem in the near future. The development and progression of DR are closely associated with the type and duration of diabetes, glucose levels, and blood pressure [1]. DR typically develops over several years and may remain asymptomatic until vision-related complications occur; it can be classified into five stages, from no apparent retinopathy to proliferative retinopathy. The disease severity scale is based on the findings of the Wisconsin Epidemiologic Study of Diabetic Retinopathy (WESDR) and the Early Treatment Diabetic Retinopathy Study (ETDRS), which allowed experts to create the International Clinical Disease Severity Scale for DR [3].

The classic early hallmark of DR is microcirculatory impairment [4, 5]. Clinical diagnosis of DR traditionally relies on the detection of microangiopathy using ophthalmoscopy, conventional fundus photography, and intravenous fluorescein angiography. Histologic studies have shown that pericyte loss and basement membrane thickening consequent to prolonged hyperglycemia characterize the onset of DR. These early changes are not visible with classic clinical imaging modalities, however, and are thus considered subclinical.

Evidence suggests that neural dysfunction in diabetes may accompany or occur before the appearance of microvascular lesions [6]. Microvascular abnormalities seen in the early-to-moderate stages of DR include loss of pericytes, basement membrane thickening, arteriole wall thickening, and formation of microaneurysms [7]. Neural degenerative changes in the retina that are associated with diabetes involve apoptosis of several populations of retinal cells, including retinal ganglion cells, which are especially sensitive to hypoxia, as well as photoreceptors and bipolar cells [5]. Neurodegenerative changes in several retinal neural cells may participate in the occurrence of the earliest morphological alternations of the vascular elements (the neurodegenerative theory of DR) [5, 8]. Defects in the retinal glial cells as a result of hyperglycemia contribute to early changes in the photoreceptors and retinal microvasculature because support for neuronal and vascular homeostasis deteriorates [9]. Consequently, DR is a disease of both retinal neurons and microcirculation. Changes in the photoreceptor layer may be found in diabetic patients before the first clinical signs of retinopathy, but it is impossible to study such changes without special imaging techniques. Advances in retinal imaging devices and development of more sophisticated optical systems can allow the capture of en face images of photoreceptors. Innovative optical technologies permit early detection of tissue changes. The adaptive optics (AO) scanning laser ophthalmoscope (AOSLO) is one noninvasive method to visualize microcirculation and retinal structures. The AO technology has been used in several studies on patients with diabetes [10–18]. Lammer et al. used high-resolution AOSLO imaging to identify changes in the human retinal cone mosaic in individuals with diabetes and across a wide range of DR severity [18]. The absolute cone density did not change significantly in diabetic eyes in this cohort, but there was increasing irregularity of cone spacing in diabetes as well as with increasing severity of DR [18].

The rtx1™ (Imagine Eyes, Orsay, France) is a microscope that uses AO technology. It permits visualization of single retinal cells (photoreceptors) and the smallest blood vessels. The image resolution achieved by this technology is comparable to a histological resolution. The AO imaging system corrects aberrations that arise from various refractive surfaces within the eye. The rtx1 camera uses infrared illumination (wavelength of 850 nm) and has a lateral resolution of 1.6 microns at 850 nm. The field of view is 4° × 4°, which corresponds to an approximately 1.2 × 1.2 mm square on the retinal surface, based on the axial length of the eye. The total image acquisition time is 4 s, during which 40 individual images can be acquired. The rtx1 microscope includes image acquisition and object recognition software for image analysis: AOdetect (for analysis of photoreceptors) and AOdetectArtery (for analysis of the retinal vasculature). The device allows the selection of any area of the retina for imaging, and measurements in the same spot can be repeated based on automatically saved coordinates [13, 16].

Several studies have demonstrated the use of the rtx1 in patients with diabetes [13, 17, 19–23]. Lombardo et al. showed with AO imaging lower cone density and abnormalities in the spatial arrangement of the parafoveal cones in DM1 patients, even when no signs of diabetic retinopathy were seen on fundoscopy [21]. The cone density decline was moderately associated with a disturbance in the glucose metabolism in diabetic patients [23]. Soliman et al. also found significantly lower cone density in patients with DR [20]. Results of AO imaging may predict anatomic or additional functional retinal outcomes in patients with diabetes.

Previous studies provided important insights into the status of photoreceptors in the eyes with DR; they did not study the status of the retinal circulation in relationship to photoreceptors. To our knowledge, there are no studies analyzing cones and retinal arteries together in the course of diabetes with the AO device and comparing these with blood test results. This study investigates the ability of an AO system to detect microvascular changes together with photoreceptor density and regularity deterioration in a cohort of diabetic subjects with mild to moderate nonproliferative DR (NPDR).

## 2. Methods

Retinal examinations with the rtx1 device were conducted between May and July 2018 at the Department of Ophthalmology, Second Faculty of Medicine, Medical University of Warsaw, located in the Ophthalmic University Hospital in Warsaw. The study protocol was approved by the Bioethical Commission of the Medical University of Warsaw. Each patient received both written and oral information explaining the objective and design of the study, the operating principles of the device, and the examination procedure. In accordance with the Declaration of Helsinki, written informed consent was obtained from all subjects who participated in the study.

### 2.1. Eligibility Criteria

The study group consisted of adult (>18 years, white European) patients recruited from Retina and Laser Services of the Ophthalmic University Hospital in Warsaw with confirmed diabetes mellitus (DM) according to the criteria of the American Diabetes Association [24] and age-matched normoglycemic healthy volunteers. Participants were divided into 2 groups: control (healthy subjects) and diabetic retinopathy (DR) study group. The control group included only healthy participants with the same exclusion criteria as the study group and a body mass index (BMI) lower than 25 kg/m^2^. The study group consisted of patients with diagnosed DR and was subdivided into mild nonproliferative diabetic retinopathy (mild NPDR) and moderate nonproliferative diabetic retinopathy (moderate NPDR) subgroups according to the International Clinical Disease Severity Scale for DR [3].

All patients provided a medical history and current blood samples to verify inclusion and exclusion criteria. The blood tests were collected two days before the retinal imaging.

The inclusion criteria for the study group were

- diagnosis of nonproliferative diabetic retinopathy in the course of diabetes mellitus
- best-corrected visual acuity equal or better than 0.4 on Snellen chart
- central fixation

The inclusion criteria for the control group were

- glycemia and blood pressure within normal limits
- no treatment for diabetes mellitus, hypertension, and coronary heart disease
- no prescription medications including oral contraception
- body mass index (BMI) less than 25 kg/m^2^
- best-corrected visual acuity equal or better than 0.4 on Snellen chart
- central fixation

The ophthalmic exclusion criteria for both groups were

- best-corrected visual acuity less than 0.4 on Snellen chart
- refractive errors for myopia > 6 diopter or astigmatism > 2.50 diopter cylindrical
- the presence or a history of glaucoma
- the presence of media opacities
- a scar, subretinal fluid, or edema in the fovea
- and no central fixation

Eligibility for study participation was confirmed by comprehensive ocular examination.

### 2.2. Patient Characteristics

An initial group of 74 patients with DR who had been seen at the laser service at the Ophthalmic Hospital from January to May 2018 was selected. Of these, 42 patients with visual acuity better or equal to 0.4 on the Snellen chart and with a diagnosis of diabetic retinopathy were invited to participate in this study. A total of 36 patients were recruited to the study group (DR group) and met the inclusion criteria. The DR group included 14 women (39%) and 22 men (61%). The mild NPDR subgroup consisted of 22 patients (61%), and moderate NPDR was diagnosed in 14 patients. Most of the patients from the study group were treated with laser photocoagulations and were under control at least 2 times a year at the offices of an ophthalmologist or diabetologist. From the study group, 12 patients had had a diagnosis of diabetes for 5 to 10 years, and 24 patients had a history of the disease for more than 10 years. Most of the diabetic patients were treated with insulin injections (32 individuals; 89%) and only 4 patients received oral treatment. Patients were asked to fill out a questionnaire about their symptoms when DM was diagnosed, their lifestyle, and coexisting diseases. Information on participants' sociodemographic characteristics, lifestyle risk factors, and medical history of diabetes and cardiovascular disease was obtained in a questionnaire (Table 1). Hypertension was defined as systolic blood pressure ≥ 140 mmHg, diastolic blood pressure ≥ 90 mmHg, or self-reported physician diagnosis of hypertension or current use of antihypertensive medications. Duration of diabetes was estimated by subtracting the year of diagnosis from the date of attendance at the research clinic. Smoking status was categorized as current, former, or never. BMI was calculated as weight in kilograms divided by the square of height in meters.

The control group consisted of 20 healthy volunteers, 13 women (65%) and 7 men (35%). Most of the participants in the control group were recruited from the hospital staff (nurses, doctors) and 4 were family members of the participants from the study group. Age and axial length were not significantly different between groups. The mean (±standard deviation) age in the control group and the DR group was 46.0 (±10.0) and 49.0 (±8.0), respectively (*p* = 0.187). The mean axial length of the right eye (OD-oculi dextri) in the control group was 23.1 ± 1.0 mm and that of the left eye (OS-oculi sinistri) was 23.1 ± 1.2 mm (Mann–Whitney *U* test, *p* = 0.580). The mean axial length of the OD in the DR group was 23.1 ± 1.1 mm and that of the OS was 23.2 ± 1.2 mm (*t*-test, *p* = 0.974). Because the eyes did not differ in terms of axial length, only the results obtained from the right eyes were included in further analyses. There were no significant differences between the eyes in both groups (*pt*-test for OD *p* = 0.974 and for OS *p* = 0.886). The characteristics of both groups are shown in Table 2.

### 2.3. Medical History of the DR Group and the Control Group

Recent results of blood parameters were analyzed, including fasting plasma glucose (FPG), glycated hemoglobin A_1c_ (HbA_1c_), triglyceride (TG), total cholesterol, low-density lipoprotein cholesterol (LDL), high-density lipoprotein cholesterol (HDL), BMI, and the correlation between the chosen parameters and cone density and retinal artery parameters.

### 2.4. Image Acquisition with the rtx1™ Adaptive Optics (AO) Camera

An AO retinal camera (rtx1™; Imagine Eyes, Orsay, France) was used to acquire images of the parafoveal cones and retinal arterioles in patients from both groups. The imaging device has 3 main components: a high-resolution fundus camera, a Shack-Hartmann wavefront sensor, and a deformable mirror for real-time correction of the aberrations of the ocular wavefront. The rtx1™ uses en face reflectance imaging with flashed, noncoherent near-infrared illumination [13].

All subjects also had noncontact ocular biometry using the IOL Master (Carl Zeiss Meditec AG, Hennigsdorf, Germany).

### 2.5. rtx1 Examination Protocol and Parameter Evaluation

Each rtx1 examination captured scans of the 4 perifoveal areas of the retina, 2° (approximately 540–600 *μ*m) off the center of the fovea (temporally, nasally, superiorly, and inferiorly) with a standardized 80 × 80 *μ*m sampling window size (fixed by the manufacturer in the new model). Most of the examinations did not require dilation of the pupils. In cases in which the width of the pupil was less than 4 mm, one drop of 1% tropicamide was administered. None of the participants from the control group required pupil dilation. Seven (19.5%) patients with NPDR had the image acquisition after 1% tropicamide drop. Study participants were instructed to fixate on the yellow fixation cross in the camera. After finding the foveal reference point of the patient, eccentricities of 2° along the meridians were measured and used for further image analyses. In most of the cases, the acquisition of the best image of cones required at least 3 scans. In retinal artery imaging, one scan was enough. It took approximately only 2-5 seconds to reach a single scan.

The best sections of images captured were selected and analyzed using the image processing and recognition software AOdetect (for analysis of photoreceptors) and AOdetectArtery (for analysis of the retinal vasculature). The analyses of the photoreceptors included the mean (±standard deviation) cone density per square millimeter of the retinal surface and the morphology of the cones in terms of neighborhood (Voronoi domain), regularity, and cone spacing. The arithmetic average of 3 measurements of the selected cone images was taken for statistical analysis.

In the analyses of the retinal vessels, 3 measurements of the retinal arteriole with a size between 70 and 130 *μ*m in the temporal superior quadrant were taken. The arithmetic average of these 3 values was taken. Measurements of the total vessel diameter (VD), the thickness of the two walls (wall 1, wall 2), and the lumen diameter (LD) were recorded, and the wall-to-lumen ratio (WLR) and the cross-sectional area of the vascular wall (WCSA) were then automatically calculated. VD resulted from a single arteriolar wall (WT) plus vessel lumen (LD) and single arteriolar wall thickness (WT): VD = WT + (WT + LD). WLR was calculated as WLR = 2 × (WT/LD). WCSA of the vascular wall was measured on the basis of VD and LD, and the value was obtained automatically from the AO artery detect software.

The area of the analyzed retina was chosen to avoid retinal vessels, hemorrhages, and laser scars during cone analysis and to avoid artery-venous crossing or close proximity of artery and vein during retinal artery analysis. We used automated cone and vessel parameter identification, in some cases with manual review of the artery profile.

### 2.6. Statistical Analyses

Descriptive analyses were conducted for all variables that were visually assessed for outliers. Three measurements of cone density in the four regions of interest (lower, upper, nasal, and temporal quadrants) were collected from each individual. The measurements were collected from the right eyes in a subset of the subjects. Kolmogorov–Smirnov and Shapiro–Wilk tests were used to determine if the parameters were normally distributed. Because the parameters were not normally distributed, we used the Mann–Whitney *U* test to compare the DR and control groups.

The Pearson correlation coefficient (*r*) was calculated to examine the linear relationship between 2 continuous variables. Statistical analyses were generated with Dell™ Statistica™ 13.1 (data analysis software system), version 12 (http://www.tibco.com).

## 3. Results

The best-corrected visual acuity (BCVA) was 1.0 in the control group, and the mean BCVA was 0.763 ± 0.151 among patients with diabetes. Most of the patients from the DR group had a BCVA better than 0.4 (29; 81%). 19% of the participants with DR had BCVA equal to 0.4.

The results of the blood test revealed significant differences between groups in the levels of white blood cells (WBC), total cholesterol, LDL cholesterol, FPG, and HbA_1c_. The results of the blood parameters for both groups are presented in Table 3.

### 3.1. Cone Parameters

**Figure 1:**
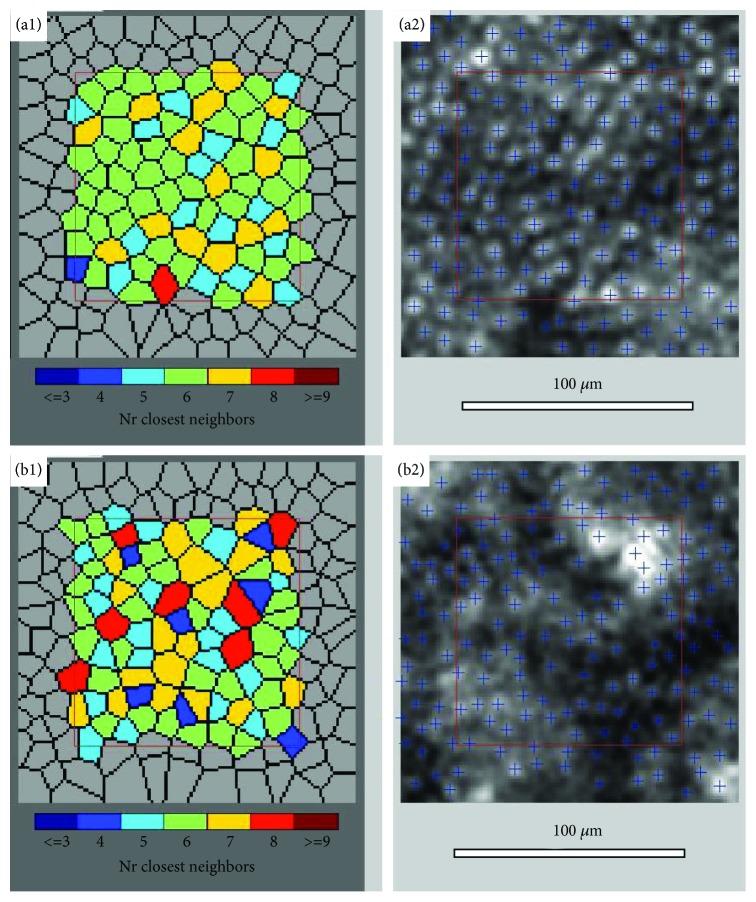
Image of the retina cones captured with the rtx1 adaptive optics retinal camera 4° × 4° degree square, visualization of cone mosaic, in a healthy patient from the control group (a1, a2) and a patient from the DR group (mild NPDR) (b1, b2).

Figure 1 shows an image of the parafoveal cone mosaic and Voronoi distribution at the 600 *μ*m eccentricity (equal to 2°) taken from a healthy patient (upper) and from a patient with mild NPDR (lower).

**Figure 2:**
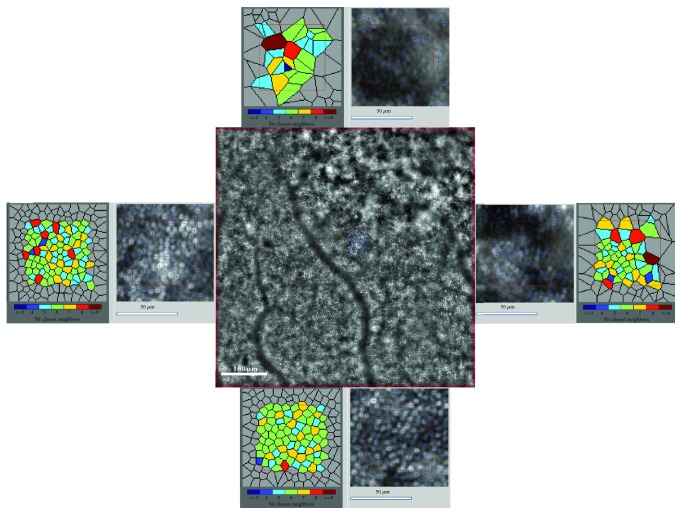
Image of the retinal cones mosaic with inset of 80 × 80 window with Voronoi representation in four quadrants captured with the rtx1 adaptive optics retinal camera, visualization of irregular cone morphology; a patient from the DR group, mild NPDR at 2° eccentricities.

Figure 2 shows irregular cone morphology taken from an eye with mild NPDR from the DR group.

The cone density was significantly lower in the DR group of patients compared to the control group at the 2° retinal eccentricities in all 4 analyzed locations. The results of cone density for all quadrants are presented in Table 4. The average (±SD) cone density, the mean of the cone density values in the 4 quadrants, in the control and DR groups were 24722 ± 3507 cells/mm^2^ and 19822 ± 4342 cells/mm^2^, respectively (*p* < 0.001; Table 4). The cone density decreased as the severity of DR increased (20440 ± 4522 cells/mm^2^ in mild NPDR, 18688 ± 3919 cells/mm^2^ in moderate NPDR; *p* 0.26). We also observed a higher standard deviation in the DR group compared to the control group (Table 4).

The interphotoreceptor spacing was significantly higher in all locations in the DR group compared to controls (Table 5).

Cone packing regularity was assessed through the analysis of Voronoi domains. The mean percentage (±SD) of cones with hexagonal Voronoi tiles (N%6) in the control and DR groups was 47.7 ± 5.9% and 42.1 ± 4.4%, respectively (*p* < 0.001; Table 5). Significant differences in cone regularity, which means the summary of the percentage of pentagonal (N%5), hexagonal (N%6), and heptagonal (N%7) cones, were found in the temporal, nasal, and superior quadrants (*p* < 0.05; Table 5). In the inferior quadrant, the regularity was also lower in the DR group (Table 5).

The mean percentage of hexagonal cones was significantly lower in both DR subgroups, but there were no significant differences between the mild NPDR (43.6 ± 4.0%) and the moderate NPDR subgroups (42.6 ± 3.6%) (*p* = 0.505; Table 5). There were also no significant differences in the other analyzed parameters of cone morphology (spacing and regularity) between the mild NPDR and moderate NPDR subgroups (Table 5).

Most of the patients with diabetes were overweight, described as BMI > 25 and <29.9, or obese, with BMI > 30. There were no significant differences in all analyzed cone and vessel parameters depending on BMI values (Table 6). We observed decreasing cone density and increasing cone spacing with higher BMI values (Table 6).

### 3.2. Retinal Artery Parameters

**Figure 3:**
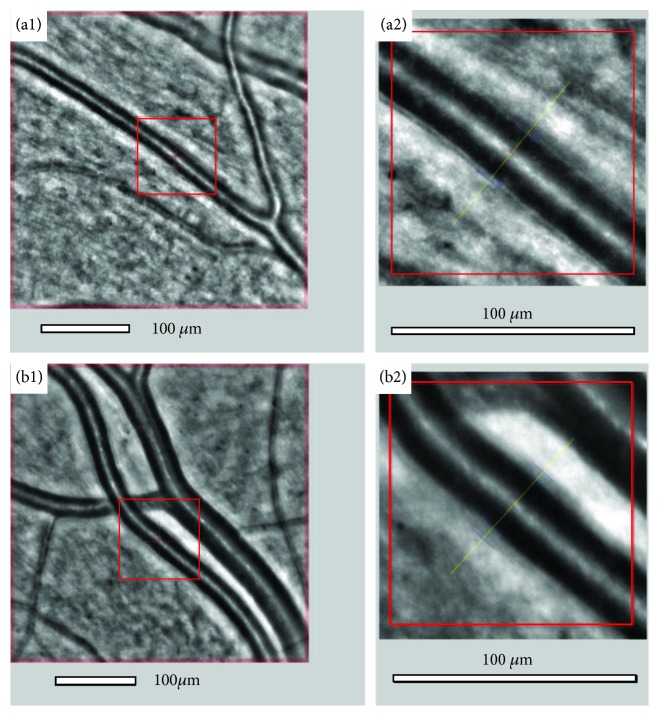
Image of the retinal artery of a patient (WLR 0.250) from the control group (a1, a2) and from the DR group with diabetes and hypertension (WLR 0.360) (b1, b2) captured with the rtx1 adaptive optics retinal camera obtained automatically, with wall and lumen visualization using AOdetectArtery.

A representative image from the retinal artery analysis is shown in Figure 3 (a1, a2). An example of microvascular changes in a diabetic patient is shown in Figure 3 (b1, b2), with increased WLR and thickening of arteriole walls.

The mean lumen and total diameter of the analyzed retinal artery were not significantly different between both groups (*p* = 0.580). The mean of both artery walls (wall1, wall 2) was significantly thicker in the DR group than in controls (*p* < 0.001). The mean WLR and WCSA values also differed significantly between the DR group and the control group (for WLR 0.339 ± 0.06 vs. 0.254 ± 0.04, *p* < 0.001; for WCSA 5567 ± 1140 vs. 4178 ± 944, respectively, *p* < 0.001; Table 7).

The lumen of the artery decreased and WLR and WCSA increased with increasing severity of DR, but there were no significant differences in all analyzed vessel parameters between the mild and moderate NPDR subgroups (Table 8).

As with the cone analysis relative to BMI values for the DR group, the analysis of retinal artery parameters revealed no significant differences in all analyzed vessel parameters based on BMI, but increasing values of all artery parameters with greater values of BMI (Table 7).

### 3.3. Correlation between Cone and Artery Parameters and Blood Parameters in the DR Group

We observed a tendency for lower values of red blood cells, hemoglobin, and hematocrit in the DR group, and the values of the two last parameters differed significantly between the two subgroups mild and moderate NPDR (Table 3).

No correlations were found between cone and artery parameters and any of the blood parameters (*p* > 0.05). There were also no correlations between cone and artery parameters and the results of the questionnaire (duration of diabetes diagnosis, cigarettes smoking, physical activity, and education level).

## 4. Discussion

In this study, we analyzed retinal photoreceptor (cone) morphology and retinal microvasculature with the new rtx1 device in a selected group of patients with DR and age-matched healthy volunteers; the rtx1 is a microscope with AO technology, which provides detailed visualization of retinal microstructures that, until recently, was only feasible in histological studies [13]. Limitations in the currently available imaging instruments focused the investigation on the microvascular changes caused by diabetes. The *in vivo* evaluation of the structure of neuronal retinal cells was not previously possible, but AO now offers a noninvasive method. We chose patients with diagnosed DR and good visual acuity, better than or equal to 0.400 on the Snellen chart. Most of the studied images were taken from patients with previous or current laser treatment, so we expected some difficulties in image acquisition. The areas of the retina selected for analysis were outside of the areas of retinal edema and laser scars. We selected the best image from several taken and made 3 measurements of cone mosaics and retinal arteries and the average value was analyzed.

Our results indicate that individuals with DR demonstrate decreased parafoveal cone density and signs of dysfunction in the retinal arterioles. Other authors presented similar results for patients with diabetes [13, 17, 20, 21, 23]. Lombardo et al. showed 6% lower cone density and differences in the percentage of hexagonal cones even in diabetic eyes without DR [21]. Soliman and coworkers also included patients with different stages of DR and found significantly lower cone density compared to the control group [20]. In our study, cone density at about 600 *μ*m off the fovea was 24722 ± 3507 in the control group, while in diabetic patients it was 19822 ± 4322. The cone density, described by Soliman et al., at the same location as ours, was comparable to our results in both the control and diabetic groups (25932 ± 2532 vs. 20528 ± 1791) [20]. We compared cone parameters across the 4 meridians and observed no significant differences. These results are similar to measures by other authors [20, 25].

The decline in cone density was observed in mild NPDR and moderate NPDR subgroups, but the differences were not significant. These results are also similar to those obtained by Soliman et al. [20]. Lammer and coworkers did not find any differences in cone density and spacing between healthy and diabetic patients [18]. They also did not notice any significant association between severity of DR and cone density and morphology [18]. Their study used adaptive optics scanning laser ophthalmoscopy (AOSLO), which may be the cause of some of the differences when comparing their results to the rtx1 analyses, while the AO technology in AOSLO is similar; the imaging modality is quite different. AOSLO rejects the scattered light greatly improving image contrast and providing more accurate cone counting. They also used a 6 mm pupil which will improve lateral resolution.

Although the absolute cone density did not change in diabetic eyes in this study, the authors observed increasing irregularity of cone spacing in the study group [18].

In our study, the interphotoreceptor distance increased significantly in the DR group compared to the control group. We also found decreasing hexagonal mosaics of cones (Voronoi 6 tiles) with increased severity of DR. The increased spacing and decreased regularity of cones in the DR group may be a result of shadowing artefacts from intraretinal pathologies, such as edema of retinal cells anterior to the photoreceptors, presence of intraretinal cysts, hemorrhages, hard exudates, and extracellular fluid accumulation [13, 18, 23, 26]. All of these pathologies may affect the reliability of our assessment of the cone mosaic. Probably as a result of this situation, there was high standard deviation in the DR group for the cone parameters compared to the healthy eyes in our study. Analyzing the retina images, we found that cone spacing regularity was disturbed by dark patches, which was also described by other authors [13, 18]. The dark patches may be caused by cone pathology, leading to decreased cone reflectance [18]. The decreased regularity and rearrangement of the cone mosaic found in our patients with DR were also described by other authors [13, 18, 21, 26]. This may support the neurodegenerative theory of DR development, in which neural cells, such as cones, degenerate in the course of diabetes [5, 8, 21, 26, 27]. Retinal neurodegeneration is also supposed to participate in microvascular changes in diabetes [5, 8].

Recently published studies of prediabetic patients or patients with diabetes but without signs of DR showed no differences in cone density and mosaic, however [22, 28]. Our findings from a previous study indicated that cone density was not affected by impaired glucose tolerance [22].

Although pathologies occur in retinal neural cells during diabetes, the clinically observed intraretinal microvascular changes serve as a basis for the classification of DR from nonproliferative to proliferative stages. Photography taken from the eyes with severe DR shows a full spectrum of microvascular abnormalities [3, 4, 7]. Recently, advances in high-resolution imaging techniques, such as AO and optical coherence tomography angiography (OCTA), have expanded our ability to visualize the living human retinal vasculature noninvasively [11–14, 19, 22].

The mean lumen and total diameter of the analyzed retinal artery were not significantly different between the control and DR groups (*p* = 0.580). Both artery walls were significantly thicker in the DR group than in controls (*p* < 0.001). The mean WLR and WCSA values also differed significantly between the DR group and the control group. The lumen of the artery decreased and the WLR and WCSA increased with increasing severity of DR, but there were no significant differences in all analyzed vessel parameters between the mild and moderate NPDR subgroups. Arterial remodeling is best characterized by increased WLR, and an overall increase in WLR can result from wall thickening, narrowing of the lumen, or a combination of both. Our results are similar to those obtained by other authors, who also described narrowing of the arteriole lumen and thickening of the arteriole walls in diabetes even without signs of retinopathy [29–35]. The results of the AusDiab study also confirmed that wider arteriolar wall caliber is a specific and effective indicator of diabetic microvascular dysfunction independent of blood pressure, glycemic control, and other retinopathy risk factors and serves as a “prepathology” marker for DR [30]. In diabetes, the lumen of retinal arterioles is narrowed due to the growth of smooth muscle cells and vascular fibrosis, a consequence of which is seen as an increased wall-to-lumen ratio [32]. Burns et al., using nonconfocal AOSLO, demonstrated results similar to ours, showing microvascular remodeling with significantly larger arteriolar wall thickness in the diabetic group compared to the controls [36]. These results support our findings of an increased WLR ratio in diabetic patients due to changes in retinal artery walls. In contrast to our results, according to the Rotterdam Study and the Blue Mountain Eye Study, a decreased arteriovenous ratio was found to depend on venular dilatation rather than arteriolar narrowing in patients with diabetes [37, 38]. The limitation of these findings is the coexistence of hypertension in the majority of diabetic patients, which may also have a strong impact on the arterial remodeling.

We analyzed the correlation of cone and retinal artery parameters with blood test results and no correlation was found. Soliman et al. presented similar results [20].

In our cohort, most of the diabetic patients were overweight, described as BMI > 25 and <29.9, or obese, with BMI > 30. There were no significant differences in all analyzed cone and vessel parameters depending on BMI values. We observed decreased cone density and increased cone spacing with greater values of BMI. The analysis of the retinal artery revealed increasing thickness of the walls, WLR, and WCSA at greater BMI values. None of these results reached the level of significance, however. To the best of our knowledge, there are no papers analyzing both cones and arteries in terms of BMI values.

Analysis of the questionnaires did not yield any correlations with cone and artery parameters. Some authors found a relationship between decreases in cone density and the duration of the disease, but others did not find such correlations [18, 20, 21].

To our knowledge, there are no studies analyzing cones and retinal arteries together in the course of diabetes with the AO devices. In some publications, primary attention is paid to changes in photoreceptors during DR, as a result of the neurodegeneration theory of DR. Others suggest a primary role for microvascular changes in DR development. The results of our work and that of other authors confirm that both cone density and retinal arterioles show early pathological changes in patients with nonproliferative diabetic retinopathy [7, 10–13, 17–23, 33, 36].

### 4.1. Study Limitations

We have identified several possible limitations of this study and the presence of some sources of bias: the high variability of cone density seen in both groups (twice as high in the DR group) and the study size and inclusion criteria (patients with good visual acuity and diabetic retinopathy). Due to our inclusion criteria, we did not check the retinas of patients at more severe stages of DR, but we decided to limit the study group because of the difficulty in obtaining good quality AO images in the eyes with many retinal changes. We wanted to be sure that the results obtained from the rtx1 would be as reliable as possible. There was also an imbalanced sex distribution across the study groups. We decided to process the study in accordance with the results of the clinical study conducted by Park et al., which found no significant differences in cone density between sexes [25]. Another limitation of this study is the inclusion of the eyes previously treated for DR; the vasculature in such eyes may not reflect the natural course of the disease, in terms of both photoreceptor mosaic and microcirculation. Lastly, imbalanced sex distribution across the study groups, small size groups, and defective AO imaging the resolution of rtx1 camera, which was used in the present study, insufficient to assess the density of extremely tightly packed cones very close to the fovea, are possible sources of bias.

This study was designed as an exploratory study without an a priori sample size calculation, and finally, high effects in outcomes of interest were observed (Tables 4, 6, and 7). Prospective studies with well-powered sample sizes are now warranted to confirm our study results. In particular, the control group seemed to be relatively small because of restricted exclusion criteria; however, sufficient statistical power (>0.80 and the post hoc tested power for the conducted study was equal to.999) to detect the DR incidence effect is relatively high.

## 5. Conclusions

In conclusion, the AO retinal imaging accurately identified the cone mosaic and retinal microvasculature in patients with diabetic retinopathy. Decreases in regularity of retinal cone arrangement and densities are consistently associated with the presence of diabetic retinopathy and increasing severity of the disease. There is a trend towards lower cone density in mild to moderate nonproliferative diabetic retinopathy. Abnormalities of the retinal arterioles found in rtx1 examinations should be considered microvascular signs of arteriolar dysfunction. These changes may be a strong risk factor for cardiovascular changes, which are also more common in this group of patients.

Further analysis on a large cohort of patients would be helpful to understand the potential of AO-based imaging biomarkers in patients with different stages of diabetic retinopathy, as well as other vascular disorders.

Analyzing microvascular changes as well as photoreceptor abnormalities could become the key for improved clinical classification of diabetic patients, better understanding of the mechanisms of diabetic retinopathy, and better patient monitoring using this technology.

## Figures and Tables

**Table 1:** Results of the questionnaire in the DR group (*n* = 36) and the control group (*n* = 20).

	No. of subjects/DR group *n* (%)	No. of subjects/control group *n* (%)
History of the DM > 5 and <10 years	**12 (33)**	**0**
History of the DM > 10 years	**24 (67)**	**0**
Elementary education	**14 (39)**	**0**
Secondary education	**16 (44)**	**6 (30)**
University education	**6 (17)**	**14 (70)**
Current cigarette smokers	**11 (31)**	**2 (10)**
Active lifestyle/sports	**13 (36)**	**12 (60)**
Diagnosis of DM after incidental blood test for glucose level	**14 (39)**	**0**
Diagnosis of DM after worrying symptoms	**22 (61)**	**0**
Family history of DM	**21 (58)**	**4 (20)**
Hypertension	**33 (92)**	**0**
Hypertension and coronary heart disease	**4 (11)**	**0**
Hypertension and neuropathy	**8 (22)**	**0**
Hypertension and nephropathy	**2 (6)**	**0**

Abbreviations: DR: diabetic retinopathy; DM: diabetes mellitus.

**Table 2:** Overall and group characteristics (*n* = 56).

	Total	Mild NPDR	Moderate NPDR	DR group	Control group	
	*n* (%)	*n* (%)	*n* (%)	*n* (%)	*n* (%)	
No. of subjects	56 (100)	22 (39)	14 (25)	36 (64)	20 (36)	
Women	27 (48.2)	8 (36)	6 (43)	14 (39)	13 (65)	
	Mean ± SD	Mean ± SD	Mean ± SD	Mean ± SD	Mean ± SD	*p* value
Age (years)	49.6 ± 8.5	51.3 ± 7.2	46.4 ± 9.5	49.0 ± 8.0	46.0 ± 10.0	0.187^†^
AL (mm)	23.2 ± 1.0	23.0 ± 1.2	23.3 ± 0.7	23.1 ± 1.1	23.1 ± 1.0	0.974^†^
BMI	28.1 ± 4.9	28.1 ± 4.5	28.2 ± 6.5	28.1 ± 5.3	24.1 ± 1.5	<0.001^‡^

^†^*t*-test; ^‡^Mann–Whitney *U* test. Abbreviations: DR: diabetic retinopathy; SD: standard deviation; AL: mean axial length; BMI: body mass index.

**Table 3:** The results of the blood parameters in both groups (*n* = 56): DR group (mild NPDR, *n* = 22; moderate NPDR, *n* = 14) and control group (*n* = 20).

Blood parameters	m ± SD^†^	m ± SD	m ± SD	m ± SD^†^	*p* value^†^ Control vs. DR	*p* value for NPDR
	Control	Mild NPDR	Moderate NPDR	DR		
WBC (k)	6.5^∗∗^ ± 1.8	7.9^‡^ ± 1.6	7.7^‡^ ± 2.0	7.8^∗∗^ ± 1.7	0.008	0.823^‡^
RBC (M)	4.7 ± 0.3	4.8^‡^ ± 0.5	4.4^‡^ ± 0.6	4.6 ± 0.5	0.838	0.068^†^
HGB (g/dl)	13.3 ± 1.4	14.2^∗†^ ± 1.3	12.9^∗†^ ± 2.0	13.7 ± 1.7	0.342	0.025^†^
HCT (%)	40.8 ± 3.3	42.8^∗†^ ± 3.3	39.1^∗†^ ± 5.5	41.4 ± 4.6	0.619	0.017^†^
PLT (k)	267 ± 75	223^†^ ± 50	239^†^ ± 76	230 ± 60	0.054	0.441^†^
FPG (mg/dl)	93^∗∗∗^ ± 7	188^†^ ± 58	168^†^ ± 55	181^∗∗∗^ ± 56	<0.001	0.314^†^
HBA_1C_ (%)	5.4^∗∗∗^ ± 0.3	8.4^†^ ± 1.7	8.8^†^ ± 2.0	8.6^∗∗∗^ ± 1.8	<0.001	0.520^†^
Total cholesterol (mg/dl)	179^∗^ ± 40	172^†^ ± 43	187^†^ ± 33	202^∗^ ± 32	0.030	0.283^†^
HDL (mg/dl)	64 ± 12	56^†^ ± 19	60^†^ ± 16	57 ± 18	0.155	0.503^†^
LDL (mg/dl)	116^∗∗^ ± 28	87^†^ ± 30	100^†^ ± 32	93^∗∗^ ± 31	0.010	0.253^†^
TG (mg/dl)	110 ± 60	136^‡^ ± 69	139^‡^ ± 69	136 ± 67	0.163	0.891^‡^

^†^*t*-test; ^‡^Mann–Whitney *U* test. Abbreviations: SD: standard deviation; DR: diabetic retinopathy; NPDR: nonproliferative diabetic retinopathy; FPG: fasting plasma glucose; HbA_1c_: glycated hemoglobin A_1c_; TG: triglyceride; LDL: low-density lipoprotein cholesterol; HDL: high-density lipoprotein cholesterol; WBC: white blood cells; RBC: red blood cells; HGB: hemoglobin; HCT: hematocrit; PLT: platelets.

**Table 4:** The mean cone density in 4 retinal quadrants at 2° eccentricities in both groups (*n* = 56) and the DR group (mild NPDR, *n* = 22; moderate NPDR, *n* = 14).

Quadrants	Mean (±SD) cone density, cone/mm^2^ DR group^a^	Mean (±SD) cone density, cone/mm^2^ Control group^b^	*p* value
T	19625 ± 4352	26478 ± 2211	<0.001^†^
N	20068 ± 4939	25586 ± 2154	<0.001^†^
S	19097 ± 4439	25387 ± 2769	<0.001^†^
I	19778 ± 4253	24923 ± 3024	<0.001^†^
4 quadrants	19822 ± 4342	24722 ± 3507	<0.001^†^
	*Mild NPDR group*	*Moderate NPDR group*	
4 quadrants	20440 ± 4522	18688 ± 3919	0.260^†^

^†^*t*-test; ^‡^Friedman ANOVA test; ^a^*p* value ^‡^for all quadrants (in column) *p* = 0.090; ^b^*p* value ^‡^for all quadrants (in column) *p* = 0.241. *p* = value for all quadrants for whole group *n* = 56 (in columns) *p* = 0.245. Abbreviations: SD: standard deviation; DR: diabetic retinopathy; NPDR: nonproliferative diabetic retinopathy; T: temporal; N: nasal; S: superior; I: inferior.

**Table 5:** The mean (±SD) spacing, Voronoi, and cone regularity in the control group and the DR group (divided into 2 subgroups: mild NPDR and moderate NPDR) in 4 quadrants.

Cone parameters in quadrants	The DR group	The control group	*p* value^†^
Spacing_ T (mm)	7.97 ± 0.88	6.80 ± 0.30	<0.001^**‡**^
Spacing_ N (mm)	7.93 ± 1.00	6.91 ± 0.30	<0.001^**‡**^
Spacing_ S (mm)	8.07 ± 0.90	6.94 ± 0.37	<0.001^**‡**^
Spacing_ I (mm)	7.92 ± 0.85	7.03 ± 0.42	<0.001^**‡**^
Spacing_4Q (mm)	7.94 ± 0.87^a^	7.08 ± 0.61^b^	<0.001^**‡**^
Voronoi N%6_T (%)	42.1 ± 4.4	47.7 ± 5.9	<0.001^†^
Voronoi N%6_N (%)	42.9 ± 5.8	47.4 ± 4.0	0.011^†^
Voronoi N%6_S (%)	44.3 ± 7.6	50.7 ± 6.1	0.008^†^
Voronoi N%6_I (%)	44.3 ± 5.4	47.6 ± 5.3	0.066^†^
Voronoi %6_4Q (%)	42.1 ± 4.4^c^	47.7 ± 5.9^d^	<0.001^†^
Regularity_T (%)	91.2 ± 3.0	93.9 ± 3.2	0.007^†^
Regularity_N (%)	90.9 ± 4.1	94.5 ± 2.6	0.005^‡^
Regularity_S (%)	75.9 ± 5.6	81.1 ± 3.6	0.003^†^
Regularity_I (%)	91.9 ± 4.1	94.3 ± 3.2	0.073^‡^
Regularity_4Q (%)	87.5 ± 3.0^e^	91.0 ± 1.7^f^	<0.001‡
*For the DR subgroups*	*Mild NPDR group*	*Moderate NPDR group*	
Spacing_4Q (mm)	7.83 ± 0.91	8.14 ± 0.79	0.214^‡^
Voronoi %6_4Q (%)	43.6 ± 4.0	42.6 ± 3.6	0.505^†^
Regularity_4Q (%)	87.8 ± 3.0	87.0 ± 3.0	0.417^‡^

^†^*t*-test; ^‡^Mann–Whitney *U* test; ^§^Friedman ANOVA test; ^a^*p* value ^§^for all quadrants (in column) *p* = 0.111; ^b^*p* value ^§^for all quadrants (in column) *p* = 0.093. ^c^*p* value ^§^for all quadrants (in column) *p* = 0.272; ^d^*p* value ^§^for all quadrants (in column) *p* = 0.946. ^c^*p* value ^§^for all quadrants (in column) *p* < 0.001; ^d^*p* value ^§^for all quadrants (in column) *p* < 0.001. Abbreviations: SD: standard deviation; T: temporal; N: nasal; S: superior; I: inferior; 4Q: for all quadrants (T, N, S, I); regularity: the summary of the percentage of pentagonal, hexagonal, and heptagonal cones in analyzed image; Voronoi_%6 tiles: percentage of hexagonal cones in analyzed image; spacing: interphotoreceptor distance.

**Table 6:** Characteristics of cone parameters in the DR group depending on BMI (*n* = 36).

Parameter	Mean (BMI < 25.0)	Mean (25.1 < BMI < 29.9)	Mean (BMI > 30.0)	*p* value KW
Cone density (1/mm^2^)	20250	17132	18788	0.172
Spacing (*μ*m)	7.7	8.6	8.1	0.204

KW: ANOVA rank Kruskal–Wallis test. Abbreviations: spacing: interphotoreceptor distance; BMI: body mass index.

**Table 7:** Characteristics of retinal artery parameters in the DR group depending on BMI (*n* = 36).

Parameter	m (BMI < 25.0)	m (25.1 < BMI < 29.9)	m (BMI > 30.0)	*p* value KW
Lumen (*μ*m)	96.5	97.0	101.8	0.725
Total diameter (*μ*m)	123.8	128.2	136.6	0.323
Wall 1 (*μ*m)	13.4	15.0	15.3	0.855
Wall 2 (*μ*m)	13.9	16.2	19.6	0.059
WCSA (*μ*m^2^)	4715	5527	6529	0.189
WLR (1)	0.285	0.332	0.343	0.554

KW-ANOVA rank Kruskal–Wallis test.

**Table 8:** Characteristics of retinal artery parameters in the DR (mild NPDR, *n* = 22; moderate NPDR, *n* = 14) and control groups (*n* = 36 and *n* = 20).

Parameters	Mean (±SD)	Mean^†^ (±SD)	Mean^†^ (±SD)	Mean (±SD)	*p* value Control vs. DR	*p* value^†^ NPDR
	Control group	Mild NPDR group	Moderate NPDR	DR group		
Lumen (*μ*m)	96.2^†^ ± 11.8	94.9 ± 13.3	93.1 ± 10.3	94.2^†^ ± 12.2	0.580^†^	0.685
Total diameter (*μ*m)	113.4 ± 32.2	127.7 ± 13.7	125.7 ± 11.8	126.9^†^ ± 12.9	0.113^†^	0.658
WALL_1 (*μ*m)	12.5^∗∗∗†^ ± 2.3	15.2 ± 3.0	15.4 ± 1.9	15.3^∗∗∗†^ ± 2.6	<0.001^†^	0.834
WALL_2 (*μ*m)	11.0^∗∗∗‡^ ± 3.5	16.0 ± 3.1	17.2 ± 3.1	16.5^∗∗∗‡^ ± 3.1	<0.001^‡^	0.295
WLR (1)	0.254^∗∗∗†^ ± 0.033	0.329 ± 0.063	0.354 ± 0.054	0.339^∗∗∗†^ ± 0.060	<0.001^†^	0.245
WCSA (*μ*m^2^)	4178^∗∗∗‡^ ± 944	5528 ± 1217	5633 ± 1041	5567^∗∗∗‡^ ± 1140	<0.001^‡^	0.796

^†^*t*-test; ^‡^Mann–Whitney *U* test. Abbreviations: SD: standard deviation; WLR: wall-to-lumen ratio; WCSA: wall cross-sectional area.

## Data Availability

The AO-RTX-DR data used to support the findings of this study are available from the corresponding author upon request.
